# Meckel-Gruber syndrome patient cells exhibit alterations in cell-substrate interaction, deformation response, and gene expression consistent with defects leading to liver fibrosis

**DOI:** 10.1186/2046-2530-4-S1-P58

**Published:** 2015-07-13

**Authors:** B Meadows, H Dawe

**Affiliations:** 1 Biosciences, Lab 211, Dawe Group, University of Exeter, Exeter, UK

## Objective

Meckel-Gruber syndrome (MKS) is a lethal ciliopathy characterised by CNS malformations, cystic kidneys, polydactyly, and liver fibrosis. Most of these can be explained by disruption of cilium-dependent signalling pathways. However, the aetiology of liver fibrosis is less well-understood. We hypothesised that alterations in cell-substrate interaction, ECM organisation, dysregulated secretion and ECM organisation are upstream of fibrosis in MKS.

## Methods

We used a combination of imaging, biophysics and RNASeq expression analysis to study alterations in cell adhesion and matrix composition in neonatal fibroblasts isolated from patients with mutations in *MKS2 *(TMEM216) or *MKS3 *(TMEM67), alongside age-matched controls.

## Results

We found that MKS cells exhibit striking differences in spreading morphology on ECM substrates in comparison to controls. While *MKS2 *cells spread faster than controls on all substrates examined, *MKS3 *cells showed a substrate-specific increase in spreading on collagen IV. We also observed differences in the morphology and distribution of focal adhesions, which appeared more mature and pervasive. Of the ~3000 genes with altered expression levels in *MKS *cells, those associated with ECM components as well as fibrosis-implicated upstream effectors of ECM organisation and cell-substrate signalling were highly over-represented. These differences were consistent with those reported in other studies of liver fibrosis. Finally, MKS cells were less resistant to deformation and fragmentation under externally applied pressure.

## Conclusions

We propose that a combination of defective regulatory signalling and excess ECM deposition, together with changes to cortical integrity and/or plasma membrane tension may contribute to the fibrotic pathology observed in patients.

